# (2*E*)-1-(2-Bromo­phen­yl)-3-(3,4,5-trimeth­oxy­phen­yl)prop-2-en-1-one

**DOI:** 10.1107/S160053681002235X

**Published:** 2010-06-16

**Authors:** Jerry P. Jasinski, Ray J. Butcher, K. Veena, B. Narayana, H. S. Yathirajan

**Affiliations:** a Department of Chemistry, Keene State College, 229 Main Street, Keene, NH 03435-2001, USA; b Department of Chemistry, Howard University, 525 College Street NW, Washington, DC 20059, USA; c Department of Studies in Chemistry, Mangalore University, Mangalagangotri, 574 199, India; d Department of Studies in Chemistry, Mangalore University, Mangalagangotri 574 199, India; e Department of Studies in Chemistry, University of Mysore, Manasagangotri, Mysore 570 006, India

## Abstract

In the chalcone title compound, C_18_H_17_BrO_4_, the dihedral angle between the mean planes of the 2-bromo- and 3,4,5-trimethoxy-substituted benzene rings is 89.3 (1)°. The angles between the mean plane of the prop-2-en-1-one group and the 2-bromo­phenyl and 3,4,5-trimeth­oxy­phenyl ring planes are 59.7 (1) and 40.5 (8)°, respectively. While no classical hydrogen bonds are present, three weak inter­molecular C—H⋯O inter­actions and weak C—H⋯Br and C—H⋯*Cg* π-ring stacking inter­actions [C—H⋯*Cg* distance = 3.377 (2) Å] are observed, which contribute to the stability of crystal packing.

## Related literature

For the radical quenching properties of included phenol groups, see: Dhar (1981 ▶). For the anti­cancer activity of chalcones, see: Dimmock *et al.* (1999 ▶). For related structures, see: Chantrapromma *et al.* (2009 ▶); Patil *et al.* (2006 ▶); Suwunwong *et al.* (2009 ▶). For bond distances and angles, see: Allen (2002 ▶).
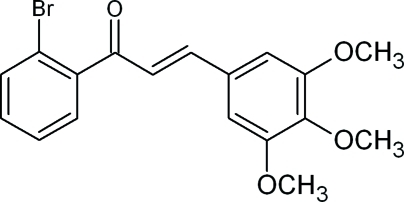


## Experimental

### 

#### Crystal data

C_18_H_17_BrO_4_
*M* *_r_* = 377.23
Orthorhombic, 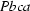

*a* = 9.9616 (4) Å
*b* = 13.6020 (13) Å
*c* = 24.4162 (17) Å
*V* = 3308.4 (4) Å^3^
*Z* = 8
Mo *K*α radiation
μ = 2.50 mm^−1^
*T* = 110 K
0.47 × 0.42 × 0.31 mm

#### Data collection

Oxford Diffraction Xcalibur diffractometer with a Ruby (Gemini Cu) detector
Absorption correction: multi-scan (*CrysAlis RED*; Oxford Diffraction, 2007 ▶) *T*_min_ = 0.499, *T*_max_ = 1.000
8122 measured reflections
3296 independent reflections
2940 reflections with *I* > 2σ(*I*)
*R*_int_ = 0.022

#### Refinement

*R*[*F* ^2^ > 2σ(*F* ^2^)] = 0.039
*wR*(*F* ^2^) = 0.112
*S* = 1.04
3296 reflections
211 parameters
H-atom parameters constrained
Δρ_max_ = 0.46 e Å^−3^
Δρ_min_ = −0.67 e Å^−3^

### 

Data collection: *CrysAlis PRO* (Oxford Diffraction, 2007 ▶); cell refinement: *CrysAlis PRO*; data reduction: *CrysAlis RED* (Oxford Diffraction, 2007 ▶); program(s) used to solve structure: *SHELXS97* (Sheldrick, 2008 ▶); program(s) used to refine structure: *SHELXL97* (Sheldrick, 2008 ▶); molecular graphics: *SHELXTL* (Sheldrick, 2008 ▶); software used to prepare material for publication: *SHELXTL*.

## Supplementary Material

Crystal structure: contains datablocks global, I. DOI: 10.1107/S160053681002235X/fj2317sup1.cif

Structure factors: contains datablocks I. DOI: 10.1107/S160053681002235X/fj2317Isup2.hkl

Additional supplementary materials:  crystallographic information; 3D view; checkCIF report

## Figures and Tables

**Table 1:** Hydrogen-bond geometry (Å, °) *Cg*2 is the centroid of the C10–C15 ring.

*D*—H⋯*A*	*D*—H	H⋯*A*	*D*⋯*A*	*D*—H⋯*A*
C6—H6*A*⋯O1^i^	0.95	2.44	3.233 (3)	140
C9—H9*A*⋯O2^ii^	0.95	2.51	3.308 (3)	141
C15—H15*A*⋯O2^ii^	0.95	2.53	3.202 (2)	128
C17—H17*C*⋯Br1^iii^	0.98	2.99	3.746 (2)	135
C17—H17*A*⋯*Cg*2^iv^	0.98	2.83	3.379 (2)	125

Symmetry codes: (i) 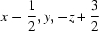; (ii) 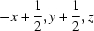; (iii) 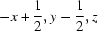; (iv) 
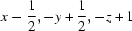
.

**Fig. 1.**
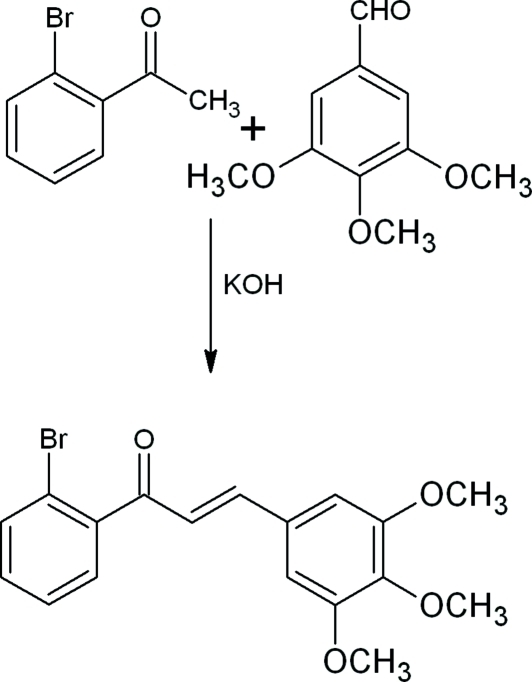
Reaction Scheme for the title compound.

**Fig. 2.**
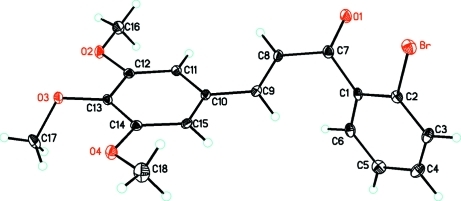
Molecular structure of (I), C18H17BrO4, showing the atom labeling scheme and 50% probability displacement ellipsoids.

**Fig. 3.**
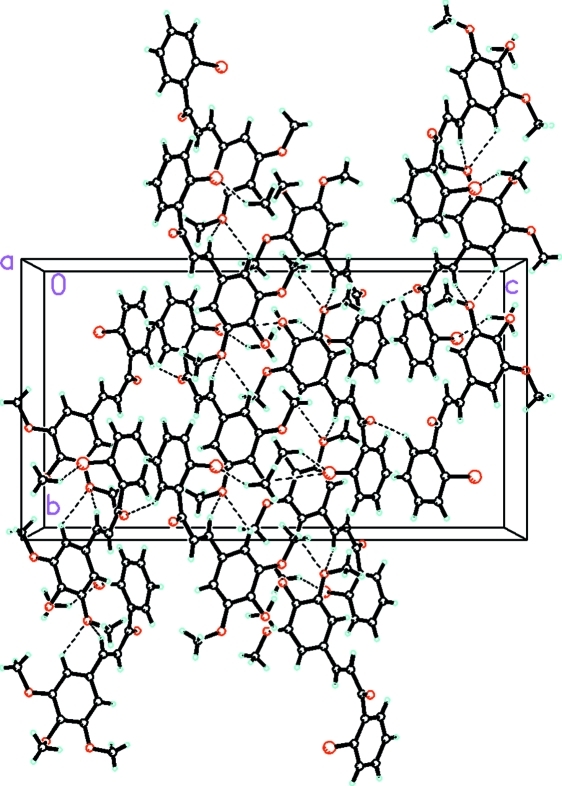
Packing diagram of the title compound, C18H17BrO4, viewed down the a axis. Dashed lines indicate weak C—H···O intermolecular hydrogen bond interactions linking the molecules into chains along the (011).

## supplementary crystallographic information

### Comment

Chalcones, or 1,3-diaryl-2-propen-1-ones, belong to the flavonoid family. Chemically they consist of open-chain flavonoids in which the two aromatic rings are joined by a three-carbon α,β-unsaturated carbonyl system. A vast number of naturally occurring chalcones are polyhydroxylated in the aryl rings. The radical quenching properties of the phenol groups present in many chalcones have raised interest in using the compounds or chalcone rich plant extracts as drugs or food preservatives (Dhar, 1981). Chalcones have been reported to possess many useful biological properties, including anti-inflammatory,antimicrobial, antifungal, antioxidant, cytotoxic, anticancer activities (Dimmock *et al.*, 1999). The crystal structures of some closely related chalcones, *viz*., (*E*)-1-(4-bromophenyl)-3-(3,4,5-trimethoxy-phenyl)prop-2-en-1-one (Suwunwong *et al.*, 2009), (*E*)-1-(4-bromophenyl)-3-(2,4,6-trimethoxyphenyl)prop-2-en-1-one (Chantrapromma *et al.*, 2009) and 1-(4-bromophenyl)-3-(2,4,5-trimethoxyphenyl)prop-2-en-1-one (Patil *et al.*, 2006) have been reported. Hence in continuation with the synthesis and crystal structure determination and also owing to the importance of these flavanoid analogs, this new bromo-trimethoxy substituted chalcone, (I), C_18_H_17_BrO_4_, is synthesized and its crystal structure is reported.

The title compound, (I), C_18_H_17_BrO_4_,is a chalcone with 2-bromophenyl and 3,4,5-trimethoxyphenyl rings bonded at opposite sides of a propene group (Fig. 2). The dihedral angle between mean planes of the benzene rings in the *ortho*-bromo and *meta*- *para*-trimethoxy substituted rings is 89.3 (1)°. The angles between the mean plane of the prop-2-ene-1-one group (C1/C7/O1/C8) and the mean planes of the benzene rings in the 2-bromophenyl (C1–C6)and 3,4,5-trimethoxyphenyl rings (C10—C15) are 59.7 (1)° and 40.5 (8)°, respectively. Bond distances and angles are in normal ranges (Allen, 2002). While no classical hydrogen bonds are present, three weak intermolecular C—H···O interactions (Fig. 3) and weak C—H···Br (Table 1) and C17—H17A···*Cg*2 π-ring stacking interactions (H17A···*Cg*2 = 2.83 Å; H17A–Perp = 2.82 Å; C17—H17A···*Cg*2 = 125°; C17···*Cg*2—H17A = 3.379 (2) Å; *Cg*2 = C10–C15) are observed which contribute to the stability of crystal packing.

### Experimental

A 50% KOH solution was added to a mixture of 2-bromo acetophenone (0.01 mol, 1.99 g) and 3,4,5-trimethoxy benzaldehyde (0.01 mol, 1.96 g) in 25 ml of ethanol (Fig. 1). The mixture was stirred for an hour at room temperature and the precipitate was collected by filtration and purified by recrystallization from ethanol. The single-crystal was grown from ethyl acetate by slow evaporation method and yield of the compound was 45% (m.p.325–327 K). Analytical data: Found (Calculated) for C_18_H_17_BrO_4_: C %: 57.26 (57.31%); H%: 4.49 (4.54%).

### Refinement

The H atoms were placed in geometrically idealized positions and constrained to ride on their parent atoms with C–H distances = 0.95–0.96Å and with *U*_iso_(H) = 1.18–1.50 *U*_eq_(C).

### Crystal data

**Table:**

C_18_H_17_BrO_4_	*F*(000) = 1536
*M**_r_* = 377.23	*D*_x_ = 1.515 Mg m^−^^3^
Orthorhombic, *P**b**c**a*	Mo *K*α radiation, λ = 0.71073 Å
Hall symbol: -P 2ac 2ab	Cell parameters from 4251 reflections
*a* = 9.9616 (4) Å	θ = 4.4–74.1°
*b* = 13.6020 (13) Å	µ = 2.50 mm^−^^1^
*c* = 24.4162 (17) Å	*T* = 110 K
*V* = 3308.4 (4) Å^3^	Chunk, colorless
*Z* = 8	0.47 × 0.42 × 0.31 mm

### Data collection

**Table:**

Oxford Diffraction Xcalibur diffractometer with a Ruby (Gemini Cu) detector	3296 independent reflections
Radiation source: Enhance (Cu) X-ray Source	2940 reflections with *I* > 2σ(*I*)
graphite	*R*_int_ = 0.022
Detector resolution: 10.5081 pixels mm^-1^	θ_max_ = 26.3°, θ_min_ = 2.6°
ω scans	*h* = −12→7
Absorption correction: multi-scan (*CrysAlis RED*; Oxford Diffraction, 2007)	*k* = −16→15
*T*_min_ = 0.499, *T*_max_ = 1.000	*l* = −30→28
8122 measured reflections	

### Refinement

**Table:**

Refinement on *F*^2^	Primary atom site location: structure-invariant direct methods
Least-squares matrix: full	Secondary atom site location: difference Fourier map
*R*[*F*^2^ > 2σ(*F*^2^)] = 0.039	Hydrogen site location: inferred from neighbouring sites
*wR*(*F*^2^) = 0.112	H-atom parameters constrained
*S* = 1.04	*w* = 1/[σ^2^(*F*_o_^2^) + (0.0769*P*)^2^ + 1.9413*P*] where *P* = (*F*_o_^2^ + 2*F*_c_^2^)/3
3296 reflections	(Δ/σ)_max_ = 0.003
211 parameters	Δρ_max_ = 0.46 e Å^−^^3^
0 restraints	Δρ_min_ = −0.67 e Å^−^^3^

### Special details

**Table:**

Experimental. IR data (KBr) \v cm^-1^: 2998 cm^-1^, 2937 cm^-1^, 2839 cm^-1^ (C—H al. str), 3058 cm^-1^ (C—H ar.str) 1646 cm^-1^ (C=O), 1580 cm^-1^ (C=C); 1245 cm^-1^ (C—O—C).
Geometry. All e.s.d.'s (except the e.s.d. in the dihedral angle between two l.s. planes) are estimated using the full covariance matrix. The cell e.s.d.'s are taken into account individually in the estimation of e.s.d.'s in distances, angles and torsion angles; correlations between e.s.d.'s in cell parameters are only used when they are defined by crystal symmetry. An approximate (isotropic) treatment of cell e.s.d.'s is used for estimating e.s.d.'s involving l.s. planes.
Refinement. Refinement of *F*^2^ against ALL reflections. The weighted *R*-factor *wR* and goodness of fit *S* are based on *F*^2^, conventional *R*-factors *R* are based on *F*, with *F* set to zero for negative *F*^2^. The threshold expression of *F*^2^ > σ(*F*^2^) is used only for calculating *R*-factors(gt) *etc*. and is not relevant to the choice of reflections for refinement. *R*-factors based on *F*^2^ are statistically about twice as large as those based on *F*, and *R*- factors based on ALL data will be even larger.

### Fractional atomic coordinates and isotropic or equivalent isotropic displacement parameters (Å^2^)

**Table:**

	*x*	*y*	*z*	*U*_iso_*/*U*_eq_	
Br1	0.74430 (2)	0.76760 (2)	0.612337 (10)	0.02659 (13)	
O1	0.74217 (17)	0.57654 (14)	0.69518 (10)	0.0340 (5)	
O2	0.31902 (16)	0.16171 (11)	0.60595 (6)	0.0191 (3)	
O3	0.17918 (15)	0.20383 (11)	0.51830 (6)	0.0180 (3)	
O4	0.14169 (16)	0.39311 (11)	0.48455 (6)	0.0203 (3)	
C1	0.5557 (2)	0.68349 (15)	0.68770 (8)	0.0159 (4)	
C2	0.6035 (2)	0.77077 (15)	0.66443 (8)	0.0169 (4)	
C3	0.5453 (2)	0.86108 (16)	0.67668 (9)	0.0219 (4)	
H3A	0.5798	0.9198	0.6610	0.026*	
C4	0.4364 (2)	0.86455 (17)	0.71200 (9)	0.0255 (5)	
H4A	0.3966	0.9260	0.7208	0.031*	
C5	0.3854 (2)	0.77852 (17)	0.73449 (9)	0.0254 (5)	
H5A	0.3101	0.7811	0.7584	0.031*	
C6	0.4441 (2)	0.68889 (16)	0.72215 (9)	0.0203 (4)	
H6A	0.4079	0.6303	0.7373	0.024*	
C7	0.6256 (2)	0.58607 (16)	0.68075 (9)	0.0196 (4)	
C8	0.5507 (2)	0.50263 (15)	0.65798 (9)	0.0183 (4)	
H8A	0.5862	0.4384	0.6630	0.022*	
C9	0.4355 (2)	0.51190 (14)	0.63061 (9)	0.0157 (4)	
H9A	0.3975	0.5758	0.6282	0.019*	
C10	0.3627 (2)	0.43154 (15)	0.60391 (8)	0.0149 (4)	
C11	0.3777 (2)	0.33362 (15)	0.62149 (8)	0.0157 (4)	
H11A	0.4324	0.3185	0.6522	0.019*	
C12	0.3114 (2)	0.25927 (15)	0.59324 (9)	0.0151 (4)	
C13	0.2319 (2)	0.28097 (16)	0.54734 (9)	0.0144 (4)	
C14	0.2176 (2)	0.37893 (15)	0.53031 (9)	0.0158 (4)	
C15	0.2808 (2)	0.45419 (15)	0.55925 (9)	0.0157 (4)	
H15A	0.2681	0.5207	0.5486	0.019*	
C16	0.3980 (2)	0.13512 (16)	0.65246 (10)	0.0242 (5)	
H16A	0.3929	0.0639	0.6581	0.036*	
H16B	0.3637	0.1690	0.6850	0.036*	
H16C	0.4916	0.1542	0.6462	0.036*	
C17	0.0357 (2)	0.19852 (18)	0.51743 (10)	0.0249 (5)	
H17A	0.0077	0.1400	0.4970	0.037*	
H17B	−0.0005	0.2574	0.4997	0.037*	
H17C	0.0018	0.1945	0.5550	0.037*	
C18	0.1511 (3)	0.48850 (18)	0.45889 (11)	0.0319 (6)	
H18A	0.0980	0.4887	0.4251	0.048*	
H18B	0.2452	0.5027	0.4502	0.048*	
H18C	0.1165	0.5388	0.4839	0.048*	

### Atomic displacement parameters (Å^2^)

**Table:**

	*U*^11^	*U*^22^	*U*^33^	*U*^12^	*U*^13^	*U*^23^
Br1	0.02410 (18)	0.0327 (2)	0.02294 (19)	−0.00535 (9)	0.00684 (8)	−0.00220 (9)
O1	0.0244 (9)	0.0223 (9)	0.0552 (13)	0.0010 (6)	−0.0201 (8)	−0.0062 (9)
O2	0.0224 (8)	0.0124 (7)	0.0226 (8)	−0.0033 (6)	−0.0055 (6)	0.0007 (6)
O3	0.0159 (7)	0.0188 (7)	0.0194 (7)	−0.0023 (6)	−0.0009 (6)	−0.0073 (6)
O4	0.0254 (8)	0.0187 (7)	0.0169 (7)	−0.0003 (6)	−0.0078 (6)	0.0006 (6)
C1	0.0177 (9)	0.0161 (10)	0.0138 (9)	−0.0046 (8)	−0.0051 (8)	−0.0020 (7)
C2	0.0177 (10)	0.0213 (11)	0.0117 (9)	−0.0025 (8)	0.0008 (8)	−0.0023 (7)
C3	0.0317 (12)	0.0159 (10)	0.0180 (10)	−0.0011 (9)	−0.0002 (9)	0.0020 (8)
C4	0.0342 (13)	0.0220 (11)	0.0204 (10)	0.0057 (10)	0.0026 (10)	−0.0045 (8)
C5	0.0251 (11)	0.0334 (13)	0.0178 (10)	−0.0009 (10)	0.0052 (9)	−0.0040 (9)
C6	0.0247 (10)	0.0207 (10)	0.0157 (10)	−0.0063 (9)	−0.0027 (8)	0.0007 (8)
C7	0.0208 (10)	0.0183 (10)	0.0197 (10)	−0.0017 (8)	−0.0051 (8)	0.0001 (8)
C8	0.0204 (10)	0.0117 (8)	0.0229 (11)	−0.0009 (8)	−0.0033 (9)	−0.0015 (8)
C9	0.0184 (10)	0.0126 (9)	0.0160 (10)	0.0005 (8)	0.0010 (8)	−0.0011 (7)
C10	0.0142 (9)	0.0141 (9)	0.0164 (9)	−0.0010 (8)	0.0018 (8)	−0.0034 (7)
C11	0.0152 (9)	0.0154 (9)	0.0163 (9)	0.0005 (8)	−0.0024 (8)	−0.0015 (8)
C12	0.0122 (9)	0.0150 (9)	0.0180 (10)	0.0000 (7)	0.0021 (8)	0.0008 (8)
C13	0.0116 (9)	0.0171 (10)	0.0144 (10)	−0.0023 (7)	0.0020 (7)	−0.0038 (8)
C14	0.0134 (8)	0.0194 (10)	0.0145 (9)	0.0019 (8)	0.0010 (8)	−0.0029 (8)
C15	0.0156 (8)	0.0133 (9)	0.0182 (10)	0.0028 (8)	0.0021 (8)	−0.0019 (8)
C16	0.0263 (11)	0.0166 (9)	0.0296 (12)	−0.0004 (9)	−0.0076 (10)	0.0053 (8)
C17	0.0171 (10)	0.0285 (12)	0.0290 (12)	−0.0073 (9)	−0.0057 (9)	0.0013 (9)
C18	0.0448 (15)	0.0260 (12)	0.0251 (12)	−0.0022 (11)	−0.0135 (11)	0.0079 (9)

### Geometric parameters (Å, °)

**Table:**

Br1—C2	1.894 (2)	C8—H8A	0.9500
O1—C7	1.221 (3)	C9—C10	1.465 (3)
O2—C12	1.365 (2)	C9—H9A	0.9500
O2—C16	1.428 (3)	C10—C15	1.396 (3)
O3—C13	1.371 (2)	C10—C11	1.407 (3)
O3—C17	1.431 (3)	C11—C12	1.391 (3)
O4—C14	1.363 (3)	C11—H11A	0.9500
O4—C18	1.444 (3)	C12—C13	1.404 (3)
C1—C6	1.396 (3)	C13—C14	1.403 (3)
C1—C2	1.400 (3)	C14—C15	1.394 (3)
C1—C7	1.506 (3)	C15—H15A	0.9500
C2—C3	1.391 (3)	C16—H16A	0.9800
C3—C4	1.386 (3)	C16—H16B	0.9800
C3—H3A	0.9500	C16—H16C	0.9800
C4—C5	1.389 (3)	C17—H17A	0.9800
C4—H4A	0.9500	C17—H17B	0.9800
C5—C6	1.385 (3)	C17—H17C	0.9800
C5—H5A	0.9500	C18—H18A	0.9800
C6—H6A	0.9500	C18—H18B	0.9800
C7—C8	1.468 (3)	C18—H18C	0.9800
C8—C9	1.334 (3)		
			
C12—O2—C16	117.25 (16)	C12—C11—C10	119.09 (19)
C13—O3—C17	115.38 (17)	C12—C11—H11A	120.5
C14—O4—C18	116.55 (17)	C10—C11—H11A	120.5
C6—C1—C2	118.09 (19)	O2—C12—C11	124.57 (19)
C6—C1—C7	118.82 (18)	O2—C12—C13	114.68 (18)
C2—C1—C7	122.91 (19)	C11—C12—C13	120.74 (19)
C3—C2—C1	121.3 (2)	O3—C13—C14	122.3 (2)
C3—C2—Br1	118.25 (16)	O3—C13—C12	117.91 (19)
C1—C2—Br1	120.35 (15)	C14—C13—C12	119.56 (19)
C4—C3—C2	119.3 (2)	O4—C14—C15	124.20 (19)
C4—C3—H3A	120.3	O4—C14—C13	115.69 (18)
C2—C3—H3A	120.3	C15—C14—C13	120.1 (2)
C3—C4—C5	120.3 (2)	C14—C15—C10	119.83 (19)
C3—C4—H4A	119.9	C14—C15—H15A	120.1
C5—C4—H4A	119.9	C10—C15—H15A	120.1
C6—C5—C4	120.0 (2)	O2—C16—H16A	109.5
C6—C5—H5A	120.0	O2—C16—H16B	109.5
C4—C5—H5A	120.0	H16A—C16—H16B	109.5
C5—C6—C1	120.9 (2)	O2—C16—H16C	109.5
C5—C6—H6A	119.5	H16A—C16—H16C	109.5
C1—C6—H6A	119.5	H16B—C16—H16C	109.5
O1—C7—C8	120.7 (2)	O3—C17—H17A	109.5
O1—C7—C1	120.0 (2)	O3—C17—H17B	109.5
C8—C7—C1	119.23 (18)	H17A—C17—H17B	109.5
C9—C8—C7	123.64 (19)	O3—C17—H17C	109.5
C9—C8—H8A	118.2	H17A—C17—H17C	109.5
C7—C8—H8A	118.2	H17B—C17—H17C	109.5
C8—C9—C10	125.33 (18)	O4—C18—H18A	109.5
C8—C9—H9A	117.3	O4—C18—H18B	109.5
C10—C9—H9A	117.3	H18A—C18—H18B	109.5
C15—C10—C11	120.61 (19)	O4—C18—H18C	109.5
C15—C10—C9	118.17 (18)	H18A—C18—H18C	109.5
C11—C10—C9	121.18 (19)	H18B—C18—H18C	109.5
			
C6—C1—C2—C3	2.4 (3)	C9—C10—C11—C12	−177.03 (19)
C7—C1—C2—C3	−172.66 (19)	C16—O2—C12—C11	1.8 (3)
C6—C1—C2—Br1	−174.42 (15)	C16—O2—C12—C13	−179.68 (19)
C7—C1—C2—Br1	10.5 (3)	C10—C11—C12—O2	179.33 (19)
C1—C2—C3—C4	−1.0 (3)	C10—C11—C12—C13	0.9 (3)
Br1—C2—C3—C4	175.89 (17)	C17—O3—C13—C14	−68.0 (3)
C2—C3—C4—C5	−0.6 (3)	C17—O3—C13—C12	117.1 (2)
C3—C4—C5—C6	0.7 (4)	O2—C12—C13—O3	−4.5 (3)
C4—C5—C6—C1	0.8 (3)	C11—C12—C13—O3	174.12 (18)
C2—C1—C6—C5	−2.3 (3)	O2—C12—C13—C14	−179.56 (18)
C7—C1—C6—C5	173.0 (2)	C11—C12—C13—C14	−1.0 (3)
C6—C1—C7—O1	−117.3 (3)	C18—O4—C14—C15	13.2 (3)
C2—C1—C7—O1	57.7 (3)	C18—O4—C14—C13	−165.9 (2)
C6—C1—C7—C8	60.9 (3)	O3—C13—C14—O4	3.4 (3)
C2—C1—C7—C8	−124.0 (2)	C12—C13—C14—O4	178.29 (18)
O1—C7—C8—C9	−164.7 (2)	O3—C13—C14—C15	−175.64 (18)
C1—C7—C8—C9	17.1 (3)	C12—C13—C14—C15	−0.8 (3)
C7—C8—C9—C10	175.4 (2)	O4—C14—C15—C10	−176.39 (19)
C8—C9—C10—C15	−153.2 (2)	C13—C14—C15—C10	2.6 (3)
C8—C9—C10—C11	24.9 (3)	C11—C10—C15—C14	−2.7 (3)
C15—C10—C11—C12	1.0 (3)	C9—C10—C15—C14	175.34 (19)

### Hydrogen-bond geometry (Å, °)

**Table:**

Cg2 is the centroid of the C10–C15 ring.

**Table:**

*D*—H···*A*	*D*—H	H···*A*	*D*···*A*	*D*—H···*A*
C6—H6A···O1^i^	0.95	2.44	3.233 (3)	140
C9—H9A···O2^ii^	0.95	2.51	3.308 (3)	141
C15—H15A···O2^ii^	0.95	2.53	3.202 (2)	128
C17—H17C···Br1^iii^	0.98	2.99	3.746 (2)	135
C17—H17A···Cg2^iv^	0.98	2.83	3.379 (2)	125

Symmetry codes: (i) *x*−1/2, *y*, −*z*+3/2; (ii) −*x*+1/2, *y*+1/2, *z*; (iii) −*x*+1/2, *y*−1/2, *z*; (iv) *x*−1/2, −*y*+1/2, −*z*+1.
